# Exercise for people with a fragility fracture of the pelvis or lower limb: a systematic review of interventions evaluated in clinical trials and reporting quality

**DOI:** 10.1186/s12891-020-03361-8

**Published:** 2020-07-04

**Authors:** David J. Keene, Colin Forde, Thavapriya Sugavanam, Mark A. Williams, Sarah E. Lamb

**Affiliations:** 1 Nuffield Department of Orthopaedics, Rheumatology and Musculoskeletal Sciences, University of Oxford, Oxford, UK; 2 Physiotherapy Department, Oxford University Hospitals NHS Foundation Trust, Oxford, UK; 3 Department of Sport, Health Sciences and Social Work, Oxford Brookes University, Oxford, UK; 4 College of Medicine and Health, University of Exeter, Exeter, UK

## Abstract

**Background:**

To aid design of exercise trials for people with pelvic and lower limb fragility fractures a systematic review was conducted to identify what types of exercise interventions and mobility outcomes have been assessed, investigate intervention reporting quality, and evaluate risk of bias in published trials.

**Methods:**

Systematic searches of electronic databases (CENTRAL, MEDLINE, EMBASE, PEDro) 1996–2019 were conducted to identify randomised controlled trials of exercise for pelvic or lower limb fragility fractures. Two reviewers independently screened titles and abstracts. One reviewer extracted data, a second verified. Two reviewers independently assessed risk of bias. Intervention reporting quality was based on TIDieR, assessed by one reviewer and verified by a second. Narrative synthesis was undertaken. Registration: PROSPERO CRD42017060905.

**Results:**

Searches identified 37 trials including 3564 participants, median sample size 81 (IQR 48–124), participants aged 81 years (IQR 79–82) and 76% (2536/3356) female. All trials focussed on people with hip fracture except one on ankle fracture. Exercise types focussed on resistance exercise in 14 trials, weight bearing exercise in 5 trials, 13 varied dose of sessions with health professionals, and 2 trials each focussed on treadmill gait training, timing of weight bearing or aerobic exercise. 30/37 (81%) of trials reported adequate sequence generation, 25/37 (68%) sufficient allocation concealment. 10/37 (27%) trials lacked outcome assessor blinding. Of 65 exercise interventions, reporting was clear for 33 (51%) in terms of when started, 61 (94%) for where delivered, 49 (75%) for who delivered, 47 (72%) for group or individual, 29 (45%) for duration, 46 (71%) for session frequency, 8 (12%) for full prescription details to enable the exercises to be reproduced, 32 (49%) clearly reported tailoring or modification, and 23 (35%) reported exercise adherence. Subjectively assessed mobility was assessed in 22/37 (59%) studies and 29/37 (78%) used an objective measure.

**Conclusions:**

All trials focussed on hip fracture, apart from one ankle fracture trial. Research into pelvic and other lower limb fragility fractures is indicated. A range of exercise types were investigated but to date deficiencies in intervention reporting hamper reproducibility. Adoption of TIDieR and CERT guidelines should improve intervention reporting as use increases. Trials would be improved by consistent blinded outcome assessor use and with consensus on which mobility outcomes should be assessed.

## Background

Fragility fractures result from low-energy trauma, usually a fall from standing height or lower. Each year 300,000 people attend UK NHS hospitals with a fragility fracture related to bone insufficiency in older age [1]. This represents a major health, social and economic problem, with an estimated annual cost of £1.8 billion [2]. Lower limb fragility fractures can have a devastating impact, resulting in mobility problems and loss of independence [3].

A core component of rehabilitation after fragility fracture is exercise prescription. A previous systematic scoping review of exercise prescription for people with any type of fragility fracture included studies up to 2009 [4]. While the scale of that review provided a comprehensive overview of exercise interventions at the time, an updated and more focussed systematic review was indicated to inform the development of future interventions for this patient group.

To the best of our knowledge no reviews to date have examined the quality of intervention reporting in trials involving people with lower limb fragility fractures. In other areas of exercise rehabilitation, limitations in reporting that prevent replication in other trials or implementation into clinical practice have been identified [5, 6]. It is therefore important to identify not only what exercise interventions have been assessed but also to establish if reporting of lower limb fragility fracture trials have similar issues in reporting quality, and if so, what areas of reporting are in greatest need of improvement to enable replicability and implementation. Exercise targets improvement in mobility after lower limb fragility fracture and this is a core outcome domain in this patient group, [7] therefore it is also important to identify what outcome measures have been used.

The overall purpose of our review was to provide evidence to guide future exercise intervention development and evaluation for people with pelvic and lower limb fragility fractures and to highlight areas of study design and intervention reporting that could be enhanced to improve the quality, replicability and implementation of future trials. Our aims were to identify the types of exercise interventions that have been tested in randomised clinical trials, investigate the reporting quality of exercise interventions, describe which mobility outcome measures have been used, and evaluate the risk of bias in the trial design and conduct.

## Methods

This systematic review was registered on the PROSPERO database (https://www.crd.york.ac.uk/prospero/display_record.php? ID = CRD42017060905) and reported according to PRISMA guidance [8].

### Eligibility

#### Types of studies

Randomised controlled trials or quasi-randomised controlled trials were considered eligible.

#### Types of participants

Studies involving adults (50 years or older) within one year of a pelvic or lower limb fracture initially treated surgically or conservatively were included. Studies were excluded if participants were younger (aged under 50 years old), unless separate data for older adults were available, or the proportion of younger adults was small (less than 10%) and, preferably, numbers balanced between the groups.

#### Types of interventions

Trials comparing different prescribed exercise regimes against each other, or prescribed exercise versus a comparator intervention such as rest, immobilisation in a brace, cast or splint, advice only, or ‘usual care’ were eligible. Exercise prescription encompassed planned physical activity, exercise or active rehabilitation prescribed by a physician, physical therapist or occupational therapist, or other allied health professional [4].

#### Types of outcomes

We extracted data on which outcome measures of mobility were used in the trials both in terms of subjectively assessed measures of mobility (e.g. Lower Extremity Functional Scale) and objective clinical measures of mobility (e.g. timed walking tests). Duration and timing of follow-up were also extracted.

### Search strategy for identification of studies

We searched the Cochrane Central Register of Controlled Trials (CENTRAL), MEDLINE, EMBASE, and the Physiotherapy Evidence Database (PEDro). We did not apply language restrictions to the searches. Studies published in 1996 or later were included. Searches were completed April 2019 and updated in MEDLINE and EMBASE in July 2019. Reference lists of included trials were checked for potentially eligible studies. An example search strategy is available in the online supplementary file.

### Selection of studies

Two reviewers independently screened the titles and abstracts using Covidence software (Covidence, Australia). We obtained full reports of potentially eligible studies, and both reviewers independently performed study selection. If agreement was not achieved by discussion at any stage, a third review author adjudicated. Articles for inclusion were limited to those written in English and published in academic journals.

### Data extraction

One author extracted data using a standard data extraction form and a second author checked the extracted data against the source while tabulating the data. The data extraction form was piloted and then modified. The following information was systematically extracted: sample size, sample demographics (age, sex, injury characteristics, time since injury), detailed descriptions of the interventions (including setting, timing, care personnel involved, training, equipment used, weight-bearing, prescription of walking aids, and the type and prescription of exercises used, and assessment of adherence), and the specified mobility outcome measures.

### Assessment of risk of bias in included studies

Two review authors independently assessed the risk of bias using Cochrane’s Risk of Bias tool [9]. We used the following domains: random sequence generation, allocation concealment, blinding of participants and personnel, blinding of outcome assessment, incomplete outcome data, selective reporting. Disagreements were resolved by discussion.

### Intervention reporting

Reporting quality for the interventions was based on the TIDieR [10] guidance for reporting complex interventions. The quality of intervention reporting was assessed by one reviewer and verified by a second reviewer. Disagreements were resolved by discussion. The criteria for the assessments are shown in Table 1.

**Table 1:** Intervention reporting assessment criteria based on TIDieR [10] recommendations

Quality assessment criteria	
**Clear**	Necessary details reported
**Unclear**	Some detail reported but did not satisfy the criteria fully
**Not reported**	No reference to the reporting domain in study report(s)
**Not applicable**	Reporting domain not applicable to the exercise intervention described
**Domain of intervention reporting**	
**When after injury**	Intended or actual timing for the start of the intervention after fracture or surgery explicitly stated.
**Where done**	Location/s of where the intervention took place. Reporting home, hospital, rehabilitation centre was sufficient.
**Who delivered**	Who administered the intervention (where applicable). Which recognised health professionals (e.g. physiotherapist/ physical therapist, occupational therapist) or for non-health professionals (e.g. administrative staff, trainer) additional information about training or expertise required.
**Group/individual**	Intervention conducted in a group, individually, or both.
**Duration of intervention**	Session duration (e.g. minutes) for supervised sessions and period of time over which intervention took place (e.g. weeks). Session duration of home programmes, and supervised sessions where only one exercise was performed and the specifics of the exercise are outlined, did not need to be reported.
**Frequency of intervention**	How often the intervention was to be completed over a specific period of time i.e. the intended schedule.
**Specifics of exercise prescription so would be reproducible**	The exercises, sets and repetitions for resistance exercises, duration for aerobic exercises, and exercise loading or intensity needed to be described in sufficient detail, or a reference provided that describes these in sufficient detail, that would allow the intervention to be reproduced.
**Tailoring/modifications**	Any component of the intervention was explicitly stated to be adapted to the individual and how this was achieved was explained.
**Adherence**	Completed intervention sessions expressed relative to the prescribed number of sessions for either the supervised or home component of the intervention where applicable e.g. 70% or 20/30, except pragmatic studies where the number of sessions was not prescribed but the number of sessions received by participants was reported.

### Data synthesis

A narrative synthesis was undertaken and interventions were grouped by exercise and fracture type. Characteristics of studies were summarised as counts and percentages for categorical data and medians with interquartile ranges for continuous data.

### Changes to protocol

The review focussed on intervention content and reporting quality as these have not been previously assessed in sufficient detail to inform the design and conduct of future trials. The originally planned focus on effectiveness and quantitative meta-analysis was not conducted as this became beyond the scope of resources for the study, and effectiveness meta-analyses are available [11].

## Results

### Study selection and characteristics

**Fig. 1:**
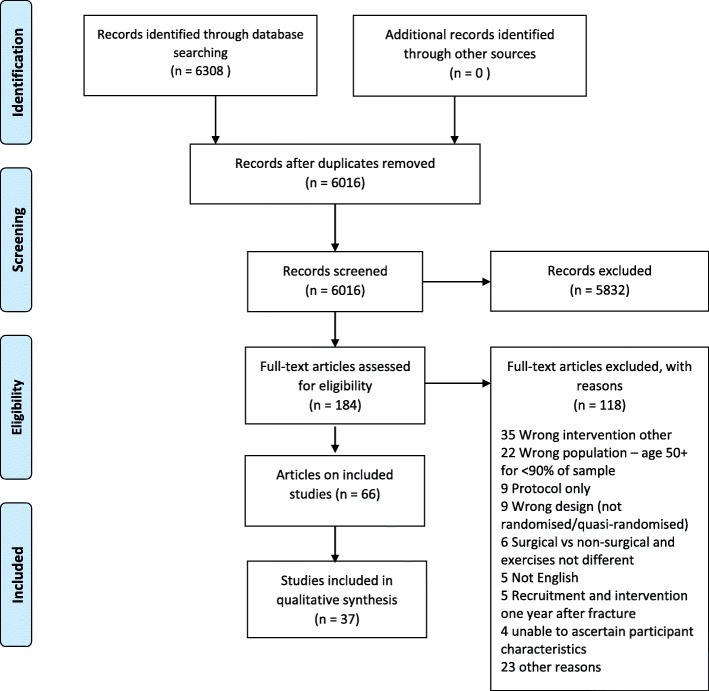
PRISMA flow diagram

Figure 1 outlines the identification, screening, and inclusion of studies. Searches identified 6308 records. After removal of duplicates, the titles and abstracts of 6016 records were screened. Of these, 184 full-text articles were assessed, and 66 articles reporting 37 trials were eligible.

### Characteristics of included studies

Of the 37 included trials, most were conducted in Australia or the USA (18/37, 49%). Trial designs were mostly parallel group (35/37, 95%) with two intervention groups (31/37, 84%), see Table 2 for detailed study characteristics. In total, 3565 participants were randomised across the 37 trials, with a median sample size of 81 (IQR 48 to 124). In 32 trials that provided adequate baseline characteristic data, participants were aged a median of 81 years (IQR 79 to 82) and 76% (2536/3356) were female. All trials focussed on people with a hip fracture except one ankle fracture trial [12] that reported results for a subgroup of participants aged more than 50 years.

**Table 2:** Study characteristics

Characteristic	
**Year published (*N*** **= 37)**	
1997–2001	3
2002–2006	12
2007–2011	9
2012–2016	11
2017–2019	2
**Country (*N*** **= 37)**	
Australia	10
USA	8
Sweden, UK	3
Norway	2
Canada, Egypt, Finland, Germany, Italy, Japan, Netherlands, Spain, Switzerland, Taiwan, Thailand	1
**RCT design (*N*** **= 37)**	
Parallel	35
Factorial	2
Cluster	0
Other	0
**Number of intervention groups (*N*** **= 37)**	
2	31
3	3
4	3
**Participants**	
Total all studies (median; IQR) (*N* = 36)	3565 (80.5; 47.5 to 123.5)
Age median (IQR) (*N* = 32)	80.75 (79.29 to 82.24)
Gender (male: female) (*N* = 32)	820: 2536
**Fracture types (*N*** **= 37)**	
Pelvic	0
Hip^a^	36
Tibia (diaphysis/metaphyseal)	0
Femur (diaphysis/distal metaphyseal)	0
Knee	0
Ankle	1
Foot	0
Mixture of lower limb fractures	0
**Orthopaedic management (*N*** **= 37)**	
Surgical	30
Conservative	0
Both	2
Unclear	5
**Exercise intervention type**^**b**^**(*N*** **= 37)**	
Resistance exercise	14
Dose of sessions with health professional	13
Weight bearing exercise	5
Treadmill training, timing of weight bearing, aerobic exercise	2
**Setting of intervention (*N*** **= 36)**	
Inpatient	11
Outpatient	6
Community	13
Combination	6
**Subjective mobility outcomes (*N*** **= 22)**	
Hip fracture studies (*N* = 21):	
Harris Hip Score^c^, Physical Performance and Mobility Examination, participant self-reported/rated mobility (not a mobility outcome questionnaire/scale)	3
Performance Oriented Mobility Assessment, Yale Physical Activity Scale, Clinician assessment of gait, Functional Ambulatory Categories	2
Functional Status Questionnaire, Hip Rating Questionnaire, Nursing Home Life-Space Diameter, Disability Rating Index, Activity Measure for Post-Acute Care, WOMAC, Assistance required for bed transfers, Modified Functional Status Index, Modified Grimby Scale, Harvard Alumni Physical Activity Index, Physical Activity Scale for the Elderly, Part C of the National Health and Nutrition Examination Survey, International Physical Activity Questionnaire long-from, Assessment of gait using 5 items from the gait component of the Performance Oriented Mobility Assessment	1
Ankle fracture studies (*N* = 1):	
Lower Extremity Functional scale, International Physical Activity Questionnaire short-form	1
**Objective mobility outcomes (*N*** **= 29)**	
Hip fracture studies (*N* = 28):	
Timed Up and Go Test	11
Gait speed	10
6 min walk test	7
Timed 6 m walk test	3
Modified Physical Performance Test, Short Physical Performance Battery, 10 m walk test, cadence during timed 6 m walk test, number of steps during timed 6 m walk test, step length during timed 6 m walk test, timed stair climbing	2
2 min walk test, 10 min walk test, 10 min walk test with obstacles, 10 min walk test with cognitive task, daily walking distance, distance walked during treatment, Lower Extremity Gain Scale, 48 h step count, timed transfer lying to sitting, 50 ft walk test, time to walk 10 ft and turn back	1
Ankle Fracture studies (*N* = 1):	
Gait speed	1
**Mixed subjective and objective mobility outcomes (*N*** **= 4)**	
Hip fracture studies (N = 4):	
Modified Iowa Level of Assistance Scale, Elderly Mobility Scale	2
Ankle Fracture studies (*N* = 0)	
**Duration of follow-up** (longest time frame in each study) (*N* = 34)	
0–6 weeks	7
> 6–16 weeks	9
> 16 weeks	18
Total median (IQR) (*N* = 33)	6 (2.5 to 12) months

^a^Three participants (1 in the control group, 2 in the intervention group) did not have a hip fracture but had elective hip surgery. These participants had a recent injurious fall

^b^ Mangione et al. 2005 is in 2 categories: *‘*Resistance exercise’ and ‘Aerobic exercise’

^c^The Harris Hip score is not categorised as a Mixed mobility outcome as the objective component of this outcome does not assess mobility

### Interventions

A range of exercise types were assessed (see Tables 2 and 3), including 14 focussing on resistance exercise, five on weight bearing exercise, 13 varied the dose of sessions with health professions, and two each focussed on treadmill training, timing of weight bearing, or aerobic exercise. These main types of intervention were often combined with other types of exercise, and compared to diverse control interventions (see Table 3).

**Table 3:** Exercise interventions and comparators across included studies

Intervention			Control		Study
**Resistance exercise**	*Resistance exercise only*				
	Resistance exercise		Aerobic exercise	Inactive control	**Mangione et al. 2005** [13] ^a^
	Resistance exercise		TENS		**Mangione et al. 2010** [14]
	Resistance exercise	Resistance exercise and supplementary nutrition	Supplementary nutrition	Advice	**Miller et al. 2006** [15]
	Resistance exercise		Inactive control		**Sherrington et al. 1997** [16]
	*Resistance and functional movement exercises*				
	Resistance and functional movement exercises		Inactive control		**Sylliaas et al. 2012** [17]
	Resistance and functional movement exercises		Inactive control		**Sylliaas et al. 2011** [18]
	*Resistance, balance, and flexibility exercise, and advice*				
	Resistance, balance, and flexibility exercise, and advice		Non-weight bearing flexibility exercise and advice		**Moseley et al. 2015** [12]
	*Resistance, functional movement, and balance exercise*				
	Resistance, functional movement, and balance exercise		Functional movement and balance exercise		**Mitchell et al. 2001** [19]
	Resistance, functional movement, and balance exercise		Physical activity and cognitive task practice		**Hauer 2002** [20]
	*Resistance and aerobic exercise*				
	Resistance and aerobic exercise	Resistance and aerobic exercise, \and behaviour change strategies	Behaviour change strategies	Inactive control	**Resnick et al 2007** [21]
	*Resistance, flexibility, balance, and aerobic exercise*				
	Resistance, flexibility, balance, and aerobic exercise		Flexibility exercise		**Binder et al. 2004** [22]
	*Resistance, aerobic, and balance exercise*				
	Resistance, aerobic, and balance exercise		Inactive control		**Peterson et al. 2004** [23]
	*Resistance and functional movement exercise, and behaviour change strategies*				
	Resistance and functional movement exercise, and behaviour change strategies		Dietary advice		**Latham et al. 2016** [24]
	*Resistance and balance exercise, and complex optional intervention components*				
	Resistance and balance exercise, and complex optional intervention components		Usual care including physiotherapy		**Singh et al. 2012** [25]
**Weight bearing exercise**	*Weight bearing resistance and functional movement exercise*				
	Weight bearing resistance and functional movement exercise		Non-weight bearing flexibility and functional movement exercise		**Sherrington et al. 2003** [26]
	Higher dose weight bearing resistance and functional movement exercise		Lower dose limited weight bearing resistance and functional movement exercise		**Moseley et al. 2009** [27]
	*Weight bearing resistance exercise only*				
	Weight bearing resistance exercise		Non-weight bearing flexibility exercise	Inactive control	**Sherrington et al. 2004** [28]
	*Weight bearing resistance and balance exercise, and advice*				
	Weight bearing resistance and balance exercise, and advice		Inactive control		**Elinge et al. 2003** [29]
	*Weight bearing balance and functional movement exercise*				
	Weight bearing balance and functional movement exercise		Limited weight bearing resistance, flexibility, and functional movement exercise		**Monticone et al. 2018** [30]
**Treadmill training**	Body Weight-Supported Treadmill Training and usual physical therapy		Usual physical therapy		**Ohoka et al. 2015** [31]
	Adaptability treadmill training and multimodal exercise^b^		Conventional treadmill training and multimodal exercise	Multimodal exercise	**van Ooijen et al. 2016** [32]
**Dose of sessions with health professional**	*Multi-disciplinary care:*				
	Higher dose multi-disciplinary care		Lower dose multi-disciplinary care		**Ryan et al. 2006** [33]
	Higher dose multi-disciplinary care		Lower dose multi-disciplinary care		**Crotty et al. 2019** [34]
	*Physiotherapy:*				
	Higher dose physiotherapy		Lower dose physiotherapy		**Kimmel et al. 2016** [35]
	Higher dose physiotherapy and high dose cholecalciferol	Higher dose physiotherapy and low dose cholecalciferol	Lower dose physiotherapy and high dose cholecalciferol	Lower dose physiotherapy and low dose cholecalciferol	**Bischoff-Ferrari et al. 2010** [36]
	Higher dose physiotherapy		Lower dose physiotherapy		**Tsauo et al. 2005** [37]
	*Occupational therapy/functional training*				
	Occupational therapy/functional training and usual care including physiotherapy		Usual care including physiotherapy		**Hagsten et al. 2004** [38]
	Occupational therapy/functional training and multimodal exercise		Multimodal exercise		**Martín-Martín et al. 2014** [39]
	Occupational therapy/functional training and higher dose multimodal exercise		Lower dose multimodal exercise		**Tinetti et al. 1999** [40]
	*Behaviour change strategies and exercise:*				
	Behaviour change strategies, unspecified exercise, and usual care		Usual care		**Suwanpasu et al. 2014** [41]
	Behaviour change strategies, higher dose functional movement exercise, and functional training		Lower dose functional movement exercise and functional training		**Zidén et al. 2008** [42]
	Behaviour change strategies, resistance, and aerobic exercise		Inactive control		**Orwig et al. 2011** [43]
	Behaviour change strategies, multimodal exercise, and flexibility exercise		Flexibility exercise		**Salpakoski et al. 2014** [44]
	Behaviour change strategies, functional movement exercise, and usual care		Usual care		**Williams et al. 2017** [45]
**Timing of weight bearing**	Early weight bearing and flexibility exercise		Delayed weight bearing and flexibility exercise		**Ali 2010** [46]
	Early weight bearing and usual physiotherapy		Delayed weight bearing and usual physiotherapy		**Oldmeadow et al. 2006** [47]
**Aerobic exercise**	*Aerobic exercise only*				
	Aerobic exercise		Resistance exercise	Inactive control	**Mangione et al. 2005** [13] ^**a**^
	*Aerobic and multimodal exercise*				
	Aerobic and multimodal exercise		Multimodal exercise		**Mendelsohn et al. 2008** [48]

Definitions: Inactive control: if the control group were not receiving any input from a healthcare clinician, or the experimental intervention commences after input from a healthcare clinician has finished for both the experimental intervention group and control group; Physiotherapy in title of control groups: if what exercise was completed as part of physiotherapy is not described; Usual care: if receiving input from healthcare clinicians but physiotherapy is not explicitly mentioned; Functional training: umbrella term to include activities of daily living practice

^a^Study appears in 2 categories: ‘Resistance exercise’ and ‘Aerobic exercise’

^b^Multimodal exercise is > 3 different types of exercise e.g. flexibility, resistance, balance, and mobility exercise

Exercises only completed in the warm-up and warm-down of exercise interventions were not included in the intervention description e.g. if flexibility exercises were only completed in the warm-up, flexibility exercise is not included in the intervention title

The setting of exercise intervention delivery was 11 for inpatients, six for outpatients, 13 for community, six were a combination, and for one trial it was unclear what the setting was.

### Outcomes

Subjectively assessed mobility outcome measures were used in 22/37 (59%) studies and 29/37 (78%) used an objective mobility measure. There were no common outcome instruments used across the trials. The most frequently used instruments were the Timed Up and Go test (11 trials) and gait speed (11 trials). The length of follow-up was a median of 6 (IQR 2.5 to 12) months.

### Risk of bias within included studies

Risk of bias assessments are shown in Table 4. Within the limitations of reporting, it was judged that 30/37 (81%) trials had adequate sequence generation and 25/37 (68%) had sufficient allocation concealment. 10/37 (27%) of trials were at high risk of bias due to a lack of outcome assessor blinding.

**Table 4:**
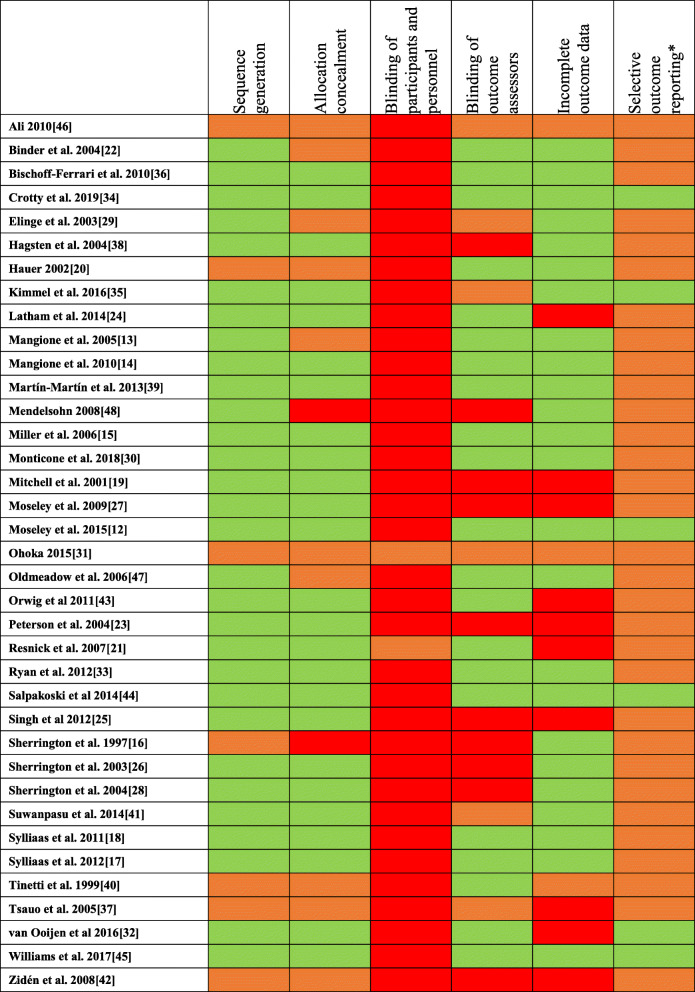
Risk of bias assessments

*(judged unclear if changes from protocol to reporting not explicitly stated or if no protocol available)

Green = low; Amber = unclear; Red = high

### Reporting quality of interventions

Of the 37 included trials there were 65 different exercise intervention groups and 16 non-exercise or inactive control comparator groups (see Table 5 for reporting quality assessments). Of the 65 exercise interventions, reporting was judged as being clearly described for 33 (51%) when treatment started after injury, 61 (94%) for where it was delivered, 49 (75%) for who delivered it, 47 (72%) on whether delivered as group or individual, 29 (45%) for the duration of the intervention, 46 (71%) for session frequency, 8 (12%) for the full prescription details to enable the intervention to be reproduced, 32 (49%) clearly reported tailoring or modification, and 23 (35%) reported exercise adherence in the trial. Of the six comparator usual care exercise interventions, only one had more than half of the intervention reporting criteria assessed as being clear.

**Table 5:**
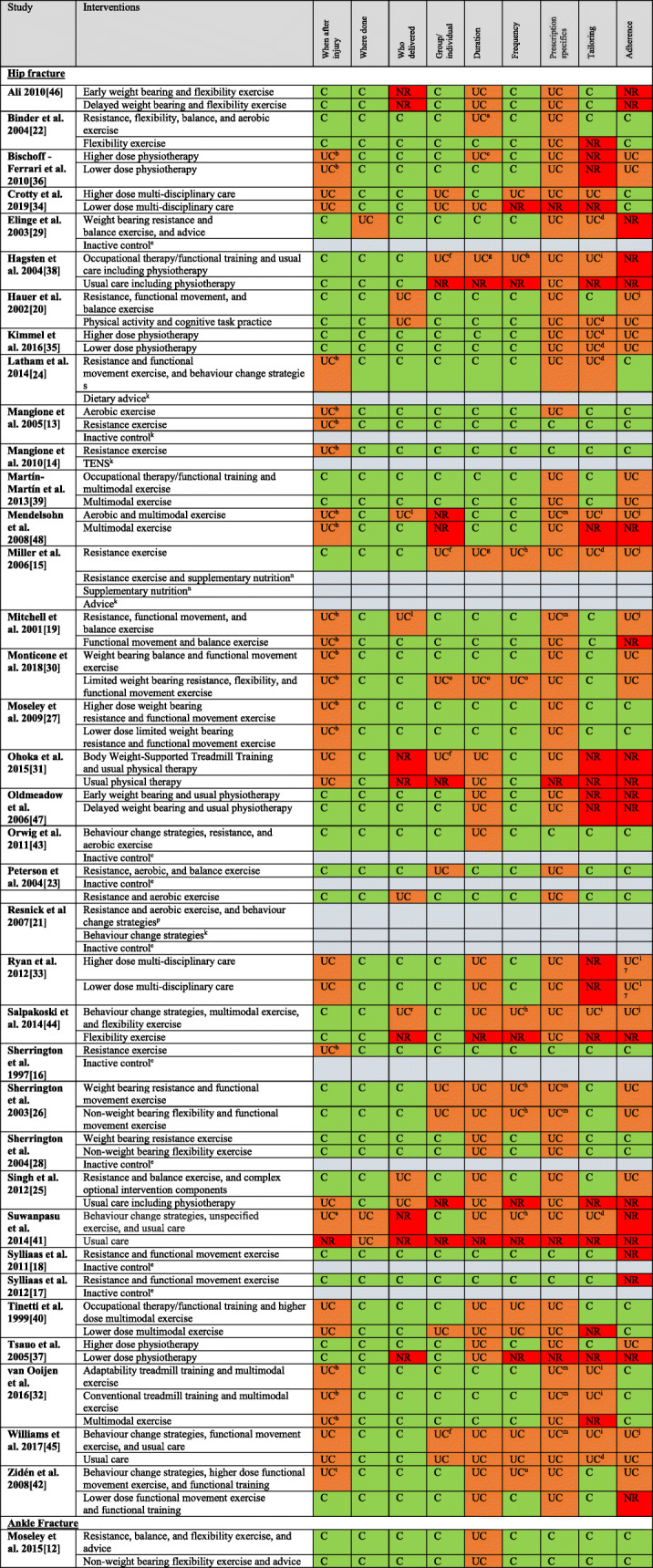
Intervention reporting assessment for each included study*

*C Clearly reported, *UC* Unclear/uncertain, *NR* Not reported, *N/A* Not applicable

^a^ Duration of phase one sessions was C, duration of phase two sessions was NR

^b^ Time from fracture/surgery to enrolment/baseline assessment/beginning of study/admission to rehab centre was C. When the intervention commenced after fracture/surgery was NR or UC

^c^ Duration of the usual component of the experimental intervention was C. Duration of the additional components of the experimental intervention was NR or UC

^d4^ Authors reported some/all of the exercise intervention was individualised but how this was achieved was NR or UC

^e^ Treatment group received no intervention

^f^ Whether the usual care component of the experimental intervention was group-based/individually completed was NR or UC. Whether the additional components of the experimental intervention were group-based/individually completed was C

^g^ Duration of usual care component of the experimental intervention was NR or UC. Duration of the additional components of the experimental intervention was C

^h^ Frequency of usual care component of the experimental intervention was NR or UC. Frequency of the additional components of the experimental intervention was C

^i^ Tailoring/modification of the usual care component of the experimental intervention was NR or UC. Tailoring/modification of the additional components of the experimental intervention was C

^j^ Adherence to usual care component of the experimental arm was NR or UC. Adherence to the additional components of the experimental intervention was C

^k^ Treatment group received an intervention that did not contain an exercise/physical activity component

^l^ Who provided usual care component of the experimental intervention was C. Who provided the additional components of the experimental intervention was NR or UC

^m^ Specifics of the usual care component of the experimental arm were NR or UC. Specifics of the additional components of the experimental intervention were C

^n^ Exercise component of this treatment group was the same as the ‘Resistance exercise’ treatment group

^o^ Contradictory information for this domain is presented in the article and the article appendix

^p^ Exercise component of this treatment group was the same as the ‘Resistance and aerobic exercise’ treatment group

^q^ Adherence to treatment was NR separately for stroke and hip fracture participants

^r^ Who provided usual care component of the experimental intervention was NR or UC. Who provided the additional components of the experimental intervention was C

^s^ Time from fracture/surgery to commencing usual care component of the experimental intervention was NR or UC. Time from fracture/surgery to commencing the additional components of the experimental intervention was C

^t^ Time from fracture/surgery to commencing the usual care component of the experimental intervention was C. Time from fracture/surgery to commencing the additional components of the experimental intervention was NR or UC

^u^ Frequency of the usual care component of the experimental intervention was C. Frequency of the additional components of the experimental intervention was NR

## Discussion

A range of exercise types have been investigated for pelvic and lower limb fragility fractures, with most trials investigating resistance exercise or higher doses of sessions with a health professional. To date deficiencies in reporting of the exercise interventions hamper reproducibility of the interventions, especially in terms of the specific details on how exercises were prescribed. Reporting of usual care exercise comparator interventions was poor. Details on exercise prescription that were most often missed related to the movements performed in the exercises, sets and repetitions for resistance exercises, duration for aerobic exercises, and exercise loading or intensity. Adoption of the TIDieR [10] checklist for reporting complex interventions should improve reporting of future trials. TIDieR was published in 2014, prior to all but five of the 37 trials included in this review. Supplementary use of the Consensus on Exercise Reporting Template (CERT) [49] is also indicated as these guidelines additionally target the main deficiencies in reporting identified in our review. It is important to recognise that the problems with exercise intervention reporting in pelvic and lower limb fragility fracture trials are consistent with other fields of rehabilitation so these issues are not isolated [5, 6].

One key area of trial design and conduct that could be improved upon in future trials is the blinding of outcome assessors as this was inadequate in 27% of trials and this could be rectified without significant additional resource burden. Blinded outcome assessors are arguably crucial given that the nature of exercise makes it self-evident what intervention is being received, as reflected in our finding that no trial had a low risk of bias assessment for blinding of participants and personnel.

With one exception, all exercise trials for adults with a pelvic or lower limb fragility fractures have been focussed on hip fracture. There is a significant burden from other non-hip fragility fractures as they often require hospitalisation and result in long-term disability, [50] therefore further research for people with pelvic and other lower limb fragility fractures is also needed. Even though most trials have focussed on hip fracture, reflecting their proportionately greater health and socio-economic impact, Sheehan and colleagues [51] have highlighted that rehabilitation trials in this patient group have underrepresented participants with cognitive impairment and nursing home residents, therefore trials focussing on other populations are also indicated.

Previous reviews have included meta-analyses to assess the effectiveness of different exercise interventions [11]. The pooling of outcomes from these trials could be problematic in the context of the intervention heterogeneity and reporting quality limitations outlined in this review. Dealing with heterogeneity in intervention components is a common challenge in quantitative synthesis of complex interventions. One approach that enables an assessment of intervention components is meta-regression, as employed by Diong and colleagues in a review of hip fracture exercise trials, [52] however, there was heterogeneity in the comparator interventions in some of the pooled studies, and there is ongoing debate as to what extent these analytical approaches manage evident clinical variations in intervention components that can interact [53].

Mobility-specific subjective and objective outcome measures were included in 59 and 78% of trials respectively but it is evident within our review that there is inconsistency in the outcome instruments used. The degree of heterogeneity in outcome measure instruments would make quantitative synthesis problematic. Further consensus work towards a core outcome set for rehabilitation trials for people with pelvic and lower limb fragility fractures would therefore be valuable.

This review has some limitations. We included English language and published literature only, meaning that some relevant studies may have been missed. Data extraction and reporting quality was not completely repeated independently by a second reviewer due to the resource limitations of the study. However, a second reviewer did verify these data against the source and any discrepancies corrected in discussion. Finally, as there was no specific intervention reporting quality assessment tool, a review specific assessment was developed drawing on the TIDieR reporting guidelines. A tool for these purposes would be valuable for future research but findings from our assessments provided some clear areas of focus for improving reporting in future exercise trials.

## Conclusion

All exercise trials for adults with a pelvic or lower limb fragility fractures have been focussed on hip fracture, apart from one ankle fracture trial. Research for people with pelvic and other lower limb fragility fractures is indicated. A wide range of exercise types have been investigated but to date deficiencies in reporting of the interventions hamper the reproducibility of the interventions, especially in terms of the specific details on how exercises were prescribed. Use of TIDieR and CERT reporting guidelines for future trials will likely improve intervention reporting. Trials of exercise interventions would also be improved by consistent use of blinded outcome assessors and with further consensus on which mobility outcomes should be assessed.

## Supplementary information

**Additional file 1.**

## Data Availability

All data generated or analysed during this study are included in this published article and its supplementary information files.

## Abbreviations

CENTRAL: Cochrane Central Register of Controlled Trials
CERT: Consensus on Exercise Reporting Template
NHS: National Health Service
TIDieR: Template for Intervention Description and Replication
