# Supplementary material for: Exercise for people with a fragility fracture of the pelvis or lower limb: a systematic review of interventions evaluated in clinical trials and reporting quality
Source: BMC Musculoskelet Disord. 2020 Jul 4;21:435. doi: 10.1186/s12891-020-03361-8 (PMC7335435; doi:10.1186/s12891-020-03361-8)
Supplement: Supplementary file 1 — Additional file 1. [file 12891_2020_3361_MOESM1_ESM.docx]

**Example search strategy**

Database: Ovid MEDLINE(R) Epub Ahead of Print, In-Process & Other Non-Indexed

Citations, Ovid MEDLINE(R) Daily and Ovid MEDLINE(R) <1946 to Present>

Search Strategy:

1 Fractures, Bone/ and Lower Extremity/

2 Ankle Fractures/

3 Femoral Fractures/

4 Tibial Fractures/

5 Pelvic Bones/ and Fractures, Bone/

6 Hip Fractures/

7 Tarsal Bones/ and Fractures, Bone/

8 Knee/ and Fractures, Bone/

9 Foot bones/ and Fractures, Bone/

10 Metatarsal Bones/ and Fractures, Bone/

11 Fractures, Bone/ and Tibia/

12 Fractures, Bone/ and Ankle/

13 Fractures, Bone/ and Femur/

14 Fractures, Bone/ and Femur Head/

15 Fractures, Bone/ and Femur Neck/

16 Femoral Neck Fractures/

17 Fractures, Bone/ and Fibula/

18 Fractures, Bone/ and Patella/

19 Fractures, Bone/ and Pubic Bone/

20 Fractures, Bone/ and Talus/

21 Fractures, Bone/ and Acetabulum/

22 Fractures, Bone/ and Sacrum/

23 Fractures, Bone/

24 (pelvis or pelvic or femur or femoral or tibia* or hip* or thigh* or ankle* or shin*

or knee* or patella or foot* or feet or "lower limb*" or "lower extremit*" or tarsal* or

metatarsal* or malleolar or malleolus or pilon or plafond or plateau or maisonneuve

or pott* or intracapsular or intertrochanter* or subtrochanter* or trochanter* or pubic

rami or pubic ramus or femoral condylar or talus or talar or acetabulum or acetabular

or sacral or sacrum or lisfranc or segond*).ti,ab.

25 23 and 24

26 (fracture* adj5 (pelvis or pelvic or femur or femoral or tibia* or hip* or thigh* or

ankle* or shin* or knee* or patella or foot* or feet or "lower limb*" or "lower extremit*"

or tarsal* or metatarsal* or malleolar or malleolus or pilon or plafond or plateau or

maisonneuve or pott* or intracapsular or intertrochanter* or subtrochanter* or

trochanter* or pubic rami or pubic ramus or femoral condylar or talus or talar or

acetabulum or acetabular or sacral or sacrum or lisfranc or segond*)).ti,ab.

27 fracture*.ti,ab.

28 ((diaphysis or diaphyseal or metaphysis or metaphyseal) adj5 (femur* or

femoral or tibia* or fibula*)).ti,ab.

29 27 and 28

30 (weber or lauge-hansen or lauge hansen).ti,ab.

31 1 or 2 or 3 or 4 or 5 or 6 or 7 or 8 or 9 or 10 or 11 or 12 or 13 or 14 or 15 or 16

or 17 or 18 or 19 or 20 or 21 or 22 or 25 or 26 or 29 or 30

32 exp Exercise Therapy/

33 (exerci* adj5 (therap* or rehab* or remed* or prescrib* or intervent*)).ti,ab.

34 (("physical activit*" or "physical exerci*" or exerci* or physiotherap* or "physical

therap*" or "occupational therap*") adj5 (therap* or program* or plan* or schedule or

visit* or rehab* or remed* or prescri* or intervent*)).ti,ab.

35 Rehabilitation/mt [Methods]

36 (power or endurance or velocity or exerci* or strength* or stretch* or mobili* or

gait or walk* or chair ris* or stair climb* or stand* or balanc* or dual task* or isokinetic

or isometric or isotonic or anaerobic or aerobic or treadmill or concentric* or

eccentric* or plyometric).ti,ab.

37 exp Exercise/

38 physical endurance/ or muscle strength/ or muscle stretching exercises/ or gait/

or walking/ or postural balance/ or early ambulation/

39 32 or 33 or 34 or 35 or 36 or 37 or 38

40 randomized controlled trial.pt.

41 controlled clinical trial.pt.

42 randomized.ab.

43 placebo.ab.

44 drug therapy.fs.

45 randomly.ab.

46 trial.ab.

47 groups.ab.

48 40 or 41 or 42 or 43 or 44 or 45 or 46 or 47

49 31 and 39 and 48

50 exp animals/ not humans.sh.

51 49 not 50

52 51

53 limit 52 to yr="1996 -Current"

54 limit 53 to yr="2017 -Current"

55 limit 51 to yr="2017-Current"

56 (fracture* adj4 (fragility or "low trauma" or "minimal trauma" or "low level" or

"low energy" or osteopor* or spontaneous*)).ti,ab.

57 39 and 48 and 56

58 exp animals/ not humans.sh.

59 57 not 58

60 limit 59 to yr="1996 -Current"

**Study characteristics**

| **Study id**  **(Country)** | **Participants**  **[Sample; Age (years mean (SD));**  **Sex (male: female)]** | **Setting of intervention** | **Intervention**  **(When, supervised/unsupervised, content, type, intensity, individual/group, frequency, duration)** | **Control**  **(When, supervised/unsupervised, content, type, intensity, individual/group, frequency, duration)** | **Functional and mobility outcomes** | **Follow-up time points** |
| --- | --- | --- | --- | --- | --- | --- |
| Hip fracture | | | | | | |
| Ali 2010  (Egypt) | N = 30 (Int: 15, Con: 15);  Age = Int: 57.93 (5.7), Con: 60.87 (4.19);  Sex = Int: 9: 6, Con: 7: 8 | Inpatients and outpatients | **Early weight bearing and flexibility exercise:**  Supervised weight bearing mobility practice of progressively greater distances and less supportive mobility aids. This commenced within 48 hours post-operatively. Participants also received supervised deep breathing, and unspecified hip and knee range of movement and strengthening exercises that commenced within 48 hours post-operatively. Treatment was individually completed, 3 sessions/week for 4 weeks. | **Delayed weight bearing and flexibility exercise**:  Supervised weight bearing mobility practice of progressively greater distances and less supportive mobility aids. This commenced 2 weeks post-operatively. Participants also received supervised deep breathing, and unspecified hip and knee range of movement and strengthening exercises that commenced within 48 hours post-operatively. Treatment was individually completed, 3 sessions/week for 4 weeks. | Subjective:  Harris Hip Score^1^ | 1 month |
| Binder et al. 2004  (USA) | N = 90 (Int: 46, Con: 44);  Age = Int: 80 (7), Con: 81 (8);  Sex = Int: 13: 33, Con: 10: 34 | Outpatients | **Resistance, flexibility, balance, and aerobic exercise:**  Commenced 103 (30) days after hip fracture and was conducted in 2 consecutive 3 month phases.  Phase 1:  Supervised flexibility, balance, aerobic (treadmill or static bike), and low intensity strengthening exercises. Exercises were adapted to target participants’ specific impairments. Group-based, 45 - 90 minutes, 3 sessions/week for 3 months.  Phase 2:  Supervised phase 1 exercises and high-intensity upper and lower limb strengthening exercises. Group-based, 3 sessions/week for 3 months. | **Flexibility exercise:**  Commenced 99 (36) days after hip fracture. Unsupervised low-intensity HEP mainly focussed on unspecified flexibility exercises. Participants could complete other forms of exercise if they wished except weights exercises. The exercise programme was performed in a supervised 1 hour group session, 1 session/month for 6 months, and as an unsupervised HEP, 3 sessions/week for 6 months. Participants also received weekly 10 minute phone calls to control for less social contact compared to the intervention group. | Subjective:  Functional Status Questionnaire, Hip Rating Questionnaire  Objective:  Modified Physical Performance Test,  gait speed | 3 & 6 months |
| Bischoff-Ferrari et al. 2010  (Switzerland) | N = 173 (Int: 87, Con: 86);  Age = Int: 83.4 (7.2), Con: 85.1 (6.5);  Sex: Int: 19: 68, Con: 17: 69  *This is combined data for both higher dose physiotherapy groups (intervention), and both Lower dose physiotherapy groups (control). | Inpatients | **Higher dose physiotherapy and high dose cholecalciferol:**  Higher dose physiotherapy component:  See below.  High dose cholecalciferol component:  See adjacent column.  **Higher dose physiotherapy and low dose cholecalciferol:**  Higher dose physiotherapy component:  Commenced during acute care, baseline was 4.2 (2.2) days after surgery. Supervised sit-to-stands, seated upper limb rowing exercise with a resistance band, balancing on 2 legs then 1, and stair climbing. At discharge participants received a written HEP of these exercises to perform unsupervised. Individually completed, 30 minutes, 7 sessions/week until discharge from acute care. The HEP was 30 minutes, 7 sessions/week. Participants also received the lower dose physiotherapy intervention.  Low dose cholecalciferol component:  See adjacent column. | **Lower dose physiotherapy and high dose cholecalciferol:**  Lower dose physiotherapy component:  See below.  High dose cholecalciferol component:  Participants received 2000 IU/day cholecalciferol.  **Lower dose physiotherapy and low dose cholecalciferol:**  Lower dose physiotherapy component:  Commenced during acute care, baseline was 4.2 (2.2) days after surgery. No details about the content of physiotherapy are provided. Individually completed, 30 minutes, 7 sessions/week until discharge from acute care.  Low dose cholecalciferol component:  Participants received 800 IU/day cholecalciferol. | Objective:  Timed Up and Go Test | 6 & 12 months |
| Crotty et al. 2019  (Australia) | N = 240 (Int: 119, Con: 121);  Age = Int: 88.6 (5.4), Con: 88.6 (5.7);  Sex = Int: 32: 87, Con: 30: 91 | Community | **Higher dose multi-disciplinary care:**  Within 24 hours of returning to nursing care facility from acute care. Supervised physiotherapy of unspecified low intensity mobility and task specific practice, muscle strengthening exercise, and training of family and nursing care facility staff. Some participants also received multi-disciplinary care from a hospital outreach team that involved a geriatrician review (n=119), dietician assessment (n=107), nurse review (n=13) and a speech pathologist review (n=5). Participants received an average of 13 physiotherapy sessions (10.6 hours) over 4 weeks.  *In addition to the intervention 73% of participants received physiotherapy. Details about the content of physiotherapy are not provided. 10 participants received a review by a dietician, and 17 participants a review by a speech pathologist arranged by the nursing care facility. | **Lower dose multi-disciplinary care:**  On return to nursing home facility from acute care. 78.5% of participants received physiotherapy with an average of 7 sessions over 4 weeks (35 minutes/week). Details about the content of physiotherapy are not provided. 15 participants received a dietetic review and 18 participants received a speech pathology review as part of usual care. | Subjective:  Nursing Home Life-Space Diameter | 1 & 12 months |
| Elinge et al. 2003  (Sweden) | N = 35 (Int: 21, Con: 14);  Age = Int: 73.1 (7.3), Con: 73.8 (11.1);  Sex = Int: 5: 16, Con: 3: 11  *There were 43 participants enrolled in the study, however complete demographic data was only available for participants who completed 3 month follow-up. This data is presented above. | Outpatients^2^ | **Weight bearing resistance exercise and advice:**  Commenced after usual care was completed, 106 - 194 days after fracture. Supervised unspecified weight bearing balance and strengthening exercise, and osteoporosis education. Group-based, 2 hours (1 hour education, 1 hour exercise), 1 session/week for 10 weeks. Participants also received an individualised HEP but no details about the content of this are provided. | **Inactive control:**  Participants in the control group received no intervention. The study intervention commenced after usual care for both groups was completed. | Nil | Immediately post intervention (10 weeks duration) & 12 months post intervention |
| Hagsten et al. 2004  (Sweden) | N = 100 (Int: 50, Con: 50);  Age = Int: 81 (range 68 -93), Con: 79 (range 65 -95);  Sex = Int: 8: 42, Con: 12: 38 | Inpatients | **Occupational therapy/functional training and usual care including physiotherapy:**  Occupational therapy/functional training component:  Commenced during acute care, 3-4 days after surgery. Supervised occupational therapy comprised of task specific training and a home visit to adapt participants’ environment prior to returning home. Individually completed, 45 - 60 minutes, 5 sessions/week, until discharge from acute care.  Usual care including physiotherapy component:  See adjacent column. | **Usual care including physiotherapy:**  Commenced during acute care, within 3-4 days after surgery. Routine post-operative care including supervised physiotherapy instruction on the use of walking aids. No further details on the content of physiotherapy are provided. | Subjective:  Disability Rating Index | On discharge & 2 months |
| Hauer et al. 2002  (Germany) | N = 28 (Int: 15, Con: 13);  Age = Int: 81.7 (7.6), Con: 80.8 (7.0);  Sex = All females  *3 participants (1 in the control group, 2 in the intervention group) did not have a hip fracture but had elective hip surgery. These participants had a recent injurious fall. | Community | **Resistance, functional movement, and balance exercise:**  Commenced immediately after discharge, mean length of stay was 23 (5) days. Supervised high intensity resistance exercise of the hip, knee extensor, and ankle plantarflexor muscles. Participants also completed mobility, and static and dynamic balance exercises. Group games, basic dancing, and tai-chi were completed if manageable for participants. Group-based, 1.5 hours of resistance exercise and 45 minutes of functional movement and balance exercise, 3 sessions/week for 12 weeks. Participants also received standard physiotherapy of massage, stretching, and ice or heat for 25 minutes, 2 sessions/week for 12 weeks. | **Physical activity and cognitive task practice:**  Commenced immediately after discharge, mean length of stay was 23 (5) days. Supervised seated physical activity and cognitive tasks that did not include resistance or balance exercises. Group-based, 1 hour, 3 sessions/week for 12 weeks. Participants also received standard physiotherapy of massage, stretching, and ice or heat for 25 minutes, 2 sessions/week for 12 weeks. | Subjective:  Performance Oriented Mobility Assessment  Objective:  Timed Up and Go Test, gait speed, timed stair climbing | 3 & 6 months |
| Kimmel et al. 2016  (Australia) | N = 92 (Int: 46, Con: 46);  Age = Int: 81.3 (7.5), Con: 81.3 (9.0);  Sex = Int: 12: 34, Con: 21: 25 | Inpatients | **Higher dose physiotherapy:** Commenced day 1 post-operatively. 1 session was with an allied health assistant and practiced the achievements of the lower dose physiotherapy session which took place in the morning (see adjacent column). 1 session was with a physiotherapist and focussed on improving function such as increasing the distance mobilised and using less supportive mobility aids**.** Individually completed, 30 minutes, 3 sessions /day, 7 days/week until discharge from acute care. | **Lower dose physiotherapy:** Commenced day 1 post-operatively and aimed to achieve independent mobility and transfers. Supervised mobility practice and bed based lower limb strengthening and range of movement exercises. Individually completed, 30 minutes, 1 session/day, 7 days/week until discharge from acute care. | Objective:  Timed Up and Go Test  Mixed:  Modified Iowa Level of Assistance Scale | Day 5 post-operatively or on discharge if this occurred earlier  *Other non-mobility related outcomes were collected at 6 months. |
| Latham et al. 2014  (USA) | N = 232 (Int: 120, Con: 112);  Age = Int: 77.2 (10.2), Con: 78.9 (9.4);  Sex = Int: 37: 83, Con: 35: 77 | Community | **Resistance and functional movement exercise, and behaviour change strategies:**  At baseline participants were 9.5 (5.2) months post fracture. Unsupervised HEP of upper and lower limb resistance, functional balance, and mobility exercises. Taught by physical therapists in 3 – 4, 1 hour home visits. Physical therapists also used behaviour change strategies to increase participation in exercise such as goal setting and using an exercise calendar to record adherence. They also telephoned participants monthly and provided a DVD of the HEP. The HEP was individually completed, 3 sessions/week for 6 months. | **Dietary advice:**  At baseline participants were 8.6 (4.8) months post fracture. Dietitians provided dietary advice during a 1 hour home visit, in 30 minute telephone calls, and by post. The frequency of contact with participants was matched to physical therapist contact with participants in the intervention group. Individually completed. | Subjective:  Activity Measure for Post-Acute Care  Objective:  Short Physical Performance Battery | 6 & 9 months |
| Mangione et al. 2005  (USA) | N = 41 (Aerobic exercise: 13, Resistance exercise: 17, Con: 11);  Age = Aerobic exercise: 79.8 (5.6), Resistance exercise: 77.9 (7.9), Con: 77.8 (7.3);  Sex = Aerobic exercise: 3: 9, Resistance exercise: 4: 7, Con: 2: 8  *There were 41 participants enrolled in the study however, complete demographic data was only reported for participants who completed follow-up. The age and sex of those that completed follow-up is presented above. | Community | **Aerobic exercise:**  The study commenced 19.7 (8.4) weeks after surgery. Supervised walking and stair climbing at 65-75% of maximum heart rate. If participants were unable to walk continuously for 20 minutes, upper and lower limb range of movement exercises were used to maintain an elevated heart rate. Individually completed, 22-23 minutes, 2 sessions/week for 8 weeks, then 1 session/week for 4 weeks.  **Resistance exercise:**  The study commenced 19.4 (11.7) weeks after surgery. Supervised high intensity open chain hip extensor, abductor, and combined hip and knee extension strengthening exercises, and single leg heel raises. Individually completed, 30-40 minutes, 2 sessions/week for 8 weeks, then 1 session/week for 4 weeks. | **Inactive control:**  Participants received written health information on non-exercise topics and were asked not to start a new exercise program until the study was finished. | Objective:  6 minute walk test,  gait speed | Aerobic exercise & Resistance exercise groups 12 weeks,  Inactive control 8 weeks |
| Mangione et al. 2010  (USA) | N = 26 (Int: 14, Con: 12);  Age = Int: 79.6 (5.9), Con: 82.0 (6.0);  Sex = Int: 2: 12, Con: 3: 9 | Community | **Resistance exercise:**  Enrolment in the study was 26 (2) weeks after fracture. Supervised high intensity open chain hip extensor, abductor, and combined hip and knee extension strengthening exercises, and single leg heel raises**.** Individually completed**,** 30 - 40 minutes, 2 sessions/week for 10 weeks. | **TENS:**  Enrolment in the study was 26 (2) weeks after fracture. Supervised TENS to the hip extensors and abductors, knee extensors, and ankle plantarflexors. Individually completed, 21 minutes, 2 sessions/week for 10 weeks. | Objective:  6 minute walk test,  gait speed, modified Physical Performance Test | 10 & 26 weeks |
| Martín-Martín et al. 2014  (Spain) | N = 122 (Int: 61, Con: 61);  Age = Int: 81.03 (5.62), Con: 83.05 (3.92);  Sex = Int: 11: 50, Con: 17: 44 | Inpatients | **Occupational therapy/functional training and multimodal exercise:**  Occupational therapy/functional training component:  Commenced the day after surgery. Supervised transfer and activities of daily living practice, advice on environmental modifications, provision of aids for activities of daily living, and provision of written information describing the training sessions for participants to review. Individually completed**,** 60 minutes for the first session, then 20 minutes sessions after this, 5 sessions/week until discharge from acute care.  Multimodal exercise component:  See adjacent column. | **Multimodal exercise:**  Commenced the day after surgery. Supervised physiotherapy involving balance, flexibility and strengthening exercises, and mobility and transfer practice. Participants did not receive any occupational therapy. Individually completed, 30 minutes, 5 sessions/week until discharge from acute care. | Subjective:  Harris Hip Score^1^ | 1, 3, & 6 months post-discharge |
| Mendelsohn et al. 2008  (Canada) | N= 20 (Int: 10, Con: 10);  Age = Int: 80.3 (7.4), Con: 81.1 (7.2);  Sex = Total: 6: 14  *The Ratio of men: women per treatment group is not reported. The total ratio of men: women is therefore presented above. | Inpatients | **Aerobic and multimodal exercise:**  Aerobic exercise component:  Baseline was 5.3 (1.5) days after fracture. Supervised arm crank ergometer exercise comprised of a 5 minute warm-up with no resistance, 20 minutes at 65% of baseline Vo_2_max, and a 5 minute cool down with no resistance. Sessions were 30 minutes, 3 sessions/week for 4 weeks.  Multimodal exercise:  See adjacent column. | **Multimodal exercise:**  Baseline was 4.9 (2.2) days after fracture. Supervised rehabilitation involving strengthening, balance, mobility and stair climbing exercises, and practice of activities of daily living. Sessions were 45 minutes, 5 sessions/week for 4 weeks. | Objective:  Timed Up and Go Test, 2 minute walk test, 10 minute walk test | 48 hours before discharge (mean length of stay was 33 days) |
| Miller et al. 2006  (Australia) | N = 100 (Resistance exercise and supplementary nutrition: 24, Resistance exercise: 25, Supplementary nutrition: 25, Advice: 26);  Age mean (95% CI) = Resistance exercise and supplementary nutrition: 82.7 (80.3 - 85.0), Resistance exercise: 84.8 (82.3 - 87.4), Advice: 83.1 (80.5 - 85.7), Supplementary nutrition: 83.5 (81.0 - 86.0);  Sex = Resistance exercise and supplementary nutrition: 7: 17, Resistance exercise: 5: 20, Supplementary nutrition 4: 21, Advice: 5: 21 | Inpatients and community | **Resistance exercise and supplementary nutrition:**  Resistance exercise component:  See below.  Supplementary nutrition component:  See adjacent column.  **Resistance exercise:**  Commenced 7 days post fracture. Supervised open chain resistance exercises of the hip extensors and abductors, and ankle dorsiflexors and plantarflexors, in supine or sitting. Individually completed, 20-30 minutes, 3 sessions/week for 12 weeks.  *All participants also received physiotherapy as part of usual care but no details on the content of this are provided. | **Supplementary nutrition:**  Commenced 7 days post fracture. Participants received an energy dense oral supplement during their acute stay and in the community.  **Advice:**  Commenced 7 days post fracture. Participants were provided with general health information such as the benefits of exercise and dietary advice, and also advised to continue with treatments provided by health professionals as part of usual care. Individually completed, 20-30 minutes, 3 sessions/week for 6 weeks, then 1 session/week for a further 6 weeks.  *All participants also received physiotherapy as part of usual care but no details on the content of this are provided. | Objective:  Gait speed | 12 weeks after commencing the interventions |
| Mitchell et al. 2001  (UK) | N = 80 (Int: 40, Con: 40);  Age = Int: 81.0 (7.59), Con: 79.1 (8.22);  Sex = Int: 6: 34, Con: 7: 33 | Inpatients | **Resistance, functional movement, and balance exercise:**  Resistance exercise component:  Participants were enrolled median (range) 15 (12-24) days post-operatively. Supervised progressive high intensity open chain knee extension strengthening exercises. Individually completed, 2 sessions/week for 6 weeks.  Functional movement and balance exercise component:  See adjacent column. | **Functional movement and balance exercise:**  Participants were enrolled median (range) 16 (13-20) days post-operatively. Supervised bed-based hip flexion and abduction range of movement exercises, standing balance exercises, bed and chair transfer practice, and mobility practice that included side stepping, backward walking and stepping over obstacles in parallel bars. Individually completed, 20 minutes, 5 sessions/week for 6 weeks. | Objective:  Timed Up and Go Test  Mixed:  Elderly Mobility Scale | 6 & 16 weeks |
| Monticone et al. 2018  (Italy) | N = 52 (Int: 26, Con: 26);  Age = Int: 77.2 (6.6), Con: 77.7 (7.5);  Sex = Int: 7: 19; Con: 8: 18 | Inpatients | **Weight bearing balance and functional movement exercise:**  Baseline was 7.9 (2.1) days after fracture. Supervised progressively challenging weight bearing balance and mobility tasks including transfer practice and stair climbing. Individually completed, 90 minutes, 5 sessions/week for 3 weeks. | **Limited weight bearing resistance, flexibility, and functional movement exercise:**  Baseline was 7.6 (2.5) days after fracture. Supervised range of movement and isometric lower limb exercises predominantly in non-weight bearing positions, mobility practice, and unspecified balance and lower limb flexibility exercises. Individually completed, 90 minutes, 5 sessions/week for 3 weeks.  *In the supplementary index sessions are reported as group-based for 1 hour, 1 session/week. This contradicts the information provided in the study report (see above). | Subjective:  Western Ontario and McMaster Universities Osteoarthritis Index (WOMAC) | 3 weeks & 12 months |
| Moseley et al. 2009  (Australia) | N = 160 (Int: 80; Con: 80);  Age = Int: 84 (8), Con: 84 (7);  Sex = Int: 15: 65, Con: 15: 65 | Inpatients and community | **Higher dose weight bearing resistance and functional movement exercise:**  Participants were admitted into a rehabilitation unit median (IQR) 14 (9-21) days after fracture. Supervised progressive weight bearing resistance exercises in standing, including step-ups, sit-to-stands, other stepping exercises, and mobility practice (using a body weight support harness if required when an inpatient). Performed initially as an inpatient, then as an unsupervised HEP with home visits of decreasing frequency. Individually completed, 60 minutes, 2 sessions/day for 16 weeks.  *All participants also completed mobility practice as part of usual post-operative care. | **Lower dose limited weight bearing resistance and functional movement exercise:**  Participants were admitted into a rehabilitation unit median (IQR) 12 (9-21) days after fracture. Supervised unspecified non-weight bearing range of movement and low intensity resistance exercises for the lower limb, and limited walking. Performed initially as an inpatient, then as an unsupervised HEP with 1 home visit/week. After 4 weeks unspecified limited weight bearing exercises were introduced and participants were advised to continue exercising independently. There were no further home visits after this point. Individually completed, 30 minutes, 7 sessions/week for 4 weeks.  *All participants also completed mobility practice as part of usual post-operative care. | Subjective:  Self-reported mobility on a likert scale, Physical Performance and Mobility Examination  Objective:  Gait speed | 4 & 16 weeks |
| Ohoka et al. 2015  (Japan) | N = 18 (Int: 9, Con: 9);  Age = NR;  Sex = All females | Inpatients | **Body Weight-Supported Treadmill Training (BWSTT) and usual physical therapy:**  BWSTT component:  Supervised BWSTT. No details about BSWTT were provided. Individually completed, 10 minutes, 4 sessions/week.  Usual physical therapy component:  See adjacent column. | **Usual physical therapy:**  No details were provided on the content of usual physical therapy. Sessions were 40 minutes, 6 sessions/week. | Objective:  Daily walking distance, 10m timed walk | At discharge, mean length of stay was 95 days |
| Oldmeadow et al. 2006  (Australia) | N = 60 (Int: 29, Con: 31);  Age = Int: 78.8 (2.14), Con: 80.0 (2.08);  Sex = Int: 8: 21, Con: 11: 20 | Inpatients | **Early weight bearing and usual physiotherapy:**  Early weight bearing component:  Supervised mobilisation ≤ 2 days post-operatively.  Usual physiotherapy component:  See adjacent column.  Treatment was individually completed, 1 session/day for 7 days.  *All participants also received usual post-operative care which included sitting out of bed as soon as possible after surgery. | **Delayed weight bearing and usual physiotherapy:**  Delayed weight bearing component:  Supervised mobilisation ≥ 3 days post-operatively.  Usual physiotherapy component:  Unspecified bed exercises, and chest physiotherapy if required.  Treatment was individually completed, 1 session/day for 7 days.  *All participants also received usual post-operative care which included sitting out of bed as soon as possible after surgery. | Subjective:  Assistance required for bed transfers  Objective:  Distance walked during treatment  Mixed:  Modified Iowa Level of Assistance Scale | 7 days post-operatively |
| Orwig et al. 2011  (USA) | N = 180 (Int: 91, Con: 89);  Age = Int: 82.5 (7.1), Con: 82.3 (6.9);  Sex = All females | Community | **Behaviour change strategies, resistance, and aerobic exercise:**  Commenced after usual physical therapy was completed, mean (range) 67.8 (25-203) days after fracture. Supervised exercise sessions with a trainer of reducing frequency which participants also performed as an unsupervised HEP. Aerobic exercise was a progressively intense stepping activity and resistance exercise was a combination of progressively intense upper and lower limb resistance exercises. Behaviour change strategies aimed to increase participant self-efficacy and included goal setting, provision of written information and an exercise calendar, and addressing barriers to exercise. Participants were also telephoned to remind them to exercise and to answer any queries. Individually completed. Trainer visits were 3 visits/week for the first 2 months, 2 visits/week for the next 2 months, then 1 visit/week, then 1 visit every second week, up to a maximum of 56 visits. Aerobic exercise was 30 minutes, 3 sessions/week for 12 months. Resistance exercise was 30 minutes, 2 sessions/week for 12 months. | **Inactive control:**  Participants in the control group received no intervention. The study intervention commenced after usual physical therapy for both groups was completed. | Subjective:  Yale Physical Activity Scale, gait analysis, modified Functional Status Index  Objective:  6 minute walk test, Lower Extremity Gain Scale | 2, 6, & 12 months post fracture |
| Peterson et al. 2004  (USA) | N = 70 (Int: 38, Con: 32);  Age = Int: 79 (7), Con: 78(8);  Sex = Int: 8: 30 Con: 4: 28  * There were 176 participants recruited overall but demographic data was only provided for participants recruited from one site (N = 70). This data is presented above. | Outpatients | **Resistance, aerobic, and balance exercise:**  Commenced after usual physical therapy care was finished, 11 (5) weeks after surgery. Supervised circuit training of hip and thigh isokinetic and isotonic resistance exercises, aerobic exercises (arm ergometer and static bike) and balancing using an unspecified ball activity. Not all exercises were completed at every session. Participants also received an individualised balance and mobility program if indicated, shown a motivational video, and given unspecified written information.  Sessions were 60 minutes, 2 sessions/week for 8 weeks. | **Inactive control:**  Participants in the control group received no intervention. The study intervention commenced after usual care for both groups was completed. | Subjective:  Gait analysis  Objective:  6 minute walk test,  Timed Up and Go Test | 14-18, 26, & 52 weeks post hospital discharge |
| Resnick et al. 2007  (USA) | N = 208 (Exercise and behaviour change: 52, Exercise: 51, Behaviour change: 54, Con: 51);  Age = (Exercise and behaviour change: 81.4 (5.8), Exercise: 82.4 (7.9), Behaviour change: 80.6 (6.9), Con: 79.7 (6.7);  Sex = All females | Community | **Resistance and aerobic exercise, and behaviour change strategies:**  Resistance and aerobic exercise component:  See below.  Behaviour change strategies component:  See adjacent column.  Both of these intervention components were administered by a trainer at the same visit.  **Resistance and aerobic exercise:**  Supervised exercise sessions with a trainer which participants also performed as an unsupervised HEP. Aerobic exercise was a progressively intense stepping activity and resistance exercise was a combination of progressively intense upper and lower limb resistance exercises. No behaviour change strategies were used. Individually completed. Trainer visits were 1 hour, initially 2 visits/week, and then 1 visit/month for the last 4 months up to a maximum of 38 visits. Aerobic exercise was 30 minutes, 3 sessions/week for 12 months. Resistance exercise was 30 minutes, 2 sessions/week for 12 months.  * All interventions commenced after usual rehabilitation services finished. The time from fracture to the first trainer visit was 28-200 days. | **Behaviour change strategies:**  Provided by trainers to increase participation in exercise and included provision of written material highlighting the benefits of exercise after a hip fracture, an exercise programme, pain management advice, goal setting and verbal encouragement. Participants did not complete exercise with trainers. Individually completed. Trainer visits were 1 hour, initially 2 visits/week, and then 1 visit/month for the last 4 months up to a maximum of 38 visits and a weekly telephone call on weeks when there were no visits.  **Inactive control:**  Participants in the control group received no intervention. The study interventions commenced after usual care for both groups was completed. | Subjective:  Yale Physical Activity Scale  Objective:  48 hour step count from Step Activity Monitor | 2, 6, & 12 months post fracture |
| Ryan et al. 2006  (UK) | N = 71 (Int: 37, Con: 34);  Age = Int: 80.7 (7.4), Con: 80.9 (6.3);  * Sex = 62: 98  *This is the ratio of males: females in the hip fracture and stroke participants combined. The sex of participants with a hip fracture per treatment group was not reported. | Community | **Higher dose multi-disciplinary care:** Baseline was 40.6 (42.2) days after fracture. Supervised multi-disciplinary (physiotherapist, occupational therapist, speech and language therapist, and therapy assistants) sessions. No details on the content of these sessions were provided. Individually completed and aimed to provide ≥ 6 sessions/week over a maximum of 12 weeks. | **Lower dose multi-disciplinary care:**  Baseline was 35 (24.6) days after fracture. Supervised multi-disciplinary (physiotherapist, occupational therapist, speech and language therapist, and therapy assistants) sessions. No details on the content of these sessions were provided. Individually completed, ≤ 3 sessions/week over a maximum of 12 weeks. | Nil | 3 months |
| Salpakoski et al. 2014  (Finland) | N = 81 (Int: 40, Con: 41);  Age = Int: 80.9 (7.7),Con: 79.1 (6.4);  Sex = Int: 9: 31, Con: 9: 32 | Community | **Behaviour change strategies, functional movement exercise, and usual care:**  Behaviour change strategies and functional movement exercise component:  Commenced on average 1 week after baseline. Baseline was 9.3 (2.3) weeks after surgery. 5-6 home sessions with a physiotherapist and an unsupervised multimodal HEP. At the first session physiotherapists implemented falls prevention strategies that included modification of participants’ home environment. At the second session a progressively challenging HEP, involving lower limb resistance, functional (mobility practice, stair climbing and reaching) and standing balance exercises, was introduced. This was reviewed 4-5 times to make it more challenging. Physiotherapists also provided advice to promote exercise adherence, in person and by follow-up phone calls. Pain management advice was also given. Individually completed. The strengthening and stretching HEP was 30 minutes, 3 sessions/week on the same day for 12 months. The balance and functional exercise HEP was 30 minutes, 2-3 sessions/week on the same day. The functional exercises were to be completed for the first 12 weeks only, balance exercises were to be completed for 12 months. Advice to promote exercise adherence was provided face-to-face at 3 and 6 months, and by telephone at 4 and 8 months, after commencing the intervention.  Usual care component:  See adjacent column. | **Usual care:**  Advice and an unsupervised HEP provided at discharge from inpatient care. The HEP usually involved range of movement exercises for the hip, knee, and ankle in supine, sitting, and/or standing. There were no resistance exercises. Some participants received no HEP and a minority receive a referral to physiotherapy**.** Usual care was individually completed. | Subjective:  Self-rated stair walking ability, modified Grimby Scale^3^  Objective:  Short Physical Performance Battery | 3, 6, 12 & 24^3^ months |
| Sherrington et al. 1997  (Australia) | N = 42 (Int: 21, Con: 21);  Age = Int: 80.0 (8.1), Con: 77.1 (8.2);  Sex = Int: 8: 13, Con: 1: 20 | Community | **Resistance exercise**:  Participants were recruited on average 7 months after fracture and completed a step-up exercise as an unsupervised HEP. Participants were advised to increase the number of repetitions completed and provided with written material to facilitate correct technique and record adherence. 1 home visit was conducted to review technique and to progress the HEP. Individually completed, at least 1 session/day for 1 month. | **Inactive control^4^:**  Participants received usual care. No details were provided on the content of usual care. | Objective:  Timed walk (mean distance 6m), cadence and number of steps during timed walk | 1 month |
| Sherrington et al. 2003  (Australia) | N = 80 (Int: 41, Con: 39);  Age = Int: 81.0 (7.0), Con: 81.1 (8.3);  Sex = Int: 14: 27, Con: 12: 27 | Inpatients | **Weight bearing resistance and functional movement exercise:**  Weight bearing resistance exercise component:  Commenced 19.2 (22.8) days after fracture. Supervised sit-to-stands, forward and lateral step-ups, stepping, and dynamic balance exercises. The exercise programme was initially completed with support from a walking frame, table, or on a tilt table if required. The exercises were progressed by increasing the repetitions, reducing support, and lowering the height of surfaces for sit-to-stands. The number of exercises performed was determined by a physiotherapist. Participants who were discharged prior to 2 week follow-up were advised to continue the programme as an unsupervised HEP. Participants received 5 sessions/week for 2 weeks.  Functional movement exercise component:  See adjacent column. | **Non-weight bearing flexibility and functional movement exercise:**  Non-weight bearing flexibility exercise component:  Commenced 17.4 (8.5) days after fracture. Supervised supine range of movement exercises of the hip, knee and ankle. If this was too challenging participants commenced with isometric contractions. The exercises were progressed by increasing the repetitions. The number of exercises performed was determined by a physiotherapist. Participants who were discharged prior to 2 week follow-up were advised to continue the programme as an unsupervised HEP. 5 sessions/week for 2 weeks. Participants received 5 sessions/week for 2 weeks.  Functional movement exercise component:  Supervised mobility practice and assessment of transfers required for discharge. | Subjective:  Physical Performance and Mobility Examination  Objective:  Timed 6m walk, steps per second for 6m walk test, step length during 6m walk test, timed transfer from lying to sitting | 2 weeks |
| Sherrington et al. 2004  (Australia) | N = 120 (Weight bearing resistance: 40, Non-weight bearing flexibility: 40, Con: 40);  Age = Weight bearing resistance: 80.1 (7.5), Non-weight bearing flexibility: 79.1 (8.9), Con: 77.2 (8.9);  Sex = Weight bearing resistance: 10: 30, Non-weight bearing flexibility 9: 31, Con: 6: 34 | Community | **Weight bearing resistance exercise:**  Commenced 153.9 (50.2) days after fracture. Unsupervised HEP of sit-to-stands, forward and lateral step-ups, stepping, and dynamic balance exercises. The HEP was initially completed using a table, chair, or mobility aid for support. The HEP was progressed by increasing the repetitions, reducing support, and lowering the height of surfaces for sit-to-stands. Individually completed, 1 session/day for 4 months. The number of exercises and repetitions to be performed by participants was determined by a physical therapist in 3 home visits. | **Non-weight bearing flexibility exercise:**  Commenced 158.5 (55.7) days after fracture. Unsupervised HEP of supine range of movement exercises of the hip, knee, and ankle. The HEP was progressed by increasing the repetitions. Individually completed, 1 session/day for 4 months. The number of exercises and repetitions to be performed by participants was determined by a physical therapist in 3 home visits.  **Inactive control:**  Participants in the control group received no intervention. | Subjective:  Physical Performance and Mobility Examination, Functional Ambulation Categories, self-reported mobility and activity levels  Objective:  Timed 6m walk test, number of steps taken for 6m walk test, timed transfer from lying to sitting | 1 & 4 months |
| Singh et al. 2012  (Australia) | N = 124 (Int: 62, Con: 62);  Age = Int: 78.4 (9), Con: 80.1 (10.1);  Sex = Int: 20: 42, Con: 19: 43 | Outpatients and community | **Resistance and balance exercise, and complex optional intervention components:**  Resistance exercise commenced 6-8 weeks after fracture. Supervised high-intensity upper and lower limb resistance exercise using resistance machines, and unspecified balance exercises. Group-based or individually completed in the outpatient department. Participants could complete a free-weight training programme at home if they were unable to attend the outpatient department. Participants also received education on preventing falls and an assessment of their home environment for falls risk. 2 sessions/week for 12 months with a monthly home visit and phone call by trainer. A variable proportion of participants also received multiple multi-disciplinary interventions at variable time points including, but not limited to, a medication review, vision assessment and treatment, nutrition assessment and treatment, provision of hip protectors and behaviour change strategies to increase self-efficacy. | **Usual care including physiotherapy:**  This involved usual multi-disciplinary care including physiotherapy as an inpatient and/or outpatient. No details about the content of physiotherapy are provided. The usual care treatment group did not receive resistance or challenging balance exercises, or the additional multi-disciplinary interventions received by the intervention group. Usual care lasted 6-12 weeks. | Subjective:  Harvard Alumni Physical Activity Index, Physical Activity Scale for the Elderly, Part C of the National Health and Nutrition Examination Survey  Objective:  6 minute walk test, gait speed | 4 & 12 months |
| Suwanpasu et al. 2014  (Thailand) | N = 46 (Int: 23, Con: 23),  Age = Int: 77.6 (7.88), Con: 72.9 (8.36);  Sex = Int: 5: 18, Con: 6: 17  *We have assumed there is an error in table 1 in the study report. We have reported the number of females in the control group as 17, based on information in the text reporting 76% of all participants were female. | Unclear | **Behaviour change strategies, unspecified exercise, and usual care:**  Behaviour change strategies and unspecified exercise component:  Comprised of 4 phases of supervised and unsupervised treatment that were completed within 7 weeks of surgery.  Phase 1:  Assessed self-efficacy and participants’ expectations of exercise.  Phase 2:  Implemented behaviour change strategies to increase self-efficacy such as provision of written material, education, and goal setting. Participants also completed unspecified exercise.  Phase 3:  Involved unspecified daily hip exercises, goal setting, and symptom management.  Phase 4:  Assessed the amount of physical activity performed.  Individually completed, 5 sessions over 7 weeks.  Usual care component:  See adjacent column. | **Usual care:**  Participants received usual care. No details on the content of usual care were provided. | Subjective:  International Physical Activity  Questionnaire (long form) | 6 weeks  Post discharge |
| Sylliaas et al. 2011  (Norway)  *Phase 1* | N = 150 (Int: 100, Con: 50);  Age = Int: 82.1 (6.5), Con: 82.9 (5.8);  Sex = Int: 15: 85, Con: 10: 40 | Outpatient | **Resistance and functional movement exercise:**  Commenced 12 weeks after fracture. A combination of supervised group-based sessions and an individually completed HEP. The group-based sessions were comprised of high intensity resisted seated knee extension, leg press, standing knee flexion, and lunge exercises. The HEP was comprised of a resisted standing knee flexion, lunging, and walking. Group-based sessions were 45 - 60 minutes, 2 sessions/week for 12 weeks. The HEP was walking for 30 minutes, 7 sessions/week if tolerated, and strengthening exercises 1 session/week for 12 weeks. | **Inactive control:**  Participants continued their normal activities. There were no restrictions on participating in exercise. | Objective:  6 minute walk test, gait speed,  Timed Up and Go Test | 12 weeks |
| Sylliaas et al. 2012  (Norway)  *Phase 2* | N= 95 (Int: 48, Con: 47);  Age = Int: 82.4 (6.5), Con: 82.2 (5.1);  Sex = Int: 9: 39, Con: 9: 38 | Outpatients | **Resistance and functional movement exercise:**  Commenced 24 weeks after fracture.  A combination of supervised group-based sessions and an individually completed HEP. The group-based sessions were comprised of high intensity resisted seated knee extension, leg press, standing knee flexion, and lunge exercises. The HEP was comprised of a resisted standing knee flexion, lunging, and walking. Group-based sessions were 45 - 60 minutes, 1 sessions/week for 12 weeks. The HEP was walking for 30 minutes, 7 sessions/week if tolerated, and strengthening exercises 1 session/week, for 12 weeks. | **Inactive control:**  Participants continued their normal activities. There were no restrictions on participating in exercise. | Objective:  6 minute walk test, gait speed,  Timed Up and Go Test | 12 weeks |
| Tinetti et al. 1999  (USA) | N = 304 (Int: 148, Con: 156);  Age = Int: 80.5 (7), Con: 79.4 (7.8);  Sex = Int: 25: 123, Con: 30: 126 | Community | **Occupational therapy/functional training and high intensity multimodal exercise:**  Participants had returned home within 100 days of surgery. Participants received supervised occupational therapy and rehabilitation nursing to optimise their performance of activities of daily living. Physical therapy was supervised and focussed on improving mobility, transfers, balance, and upper and lower limb strength. Participants’ environment was also modified and equipment provided to facilitate mobility and transfers. Participants were required to perform an unsupervised HEP of balance, and upper and lower limb strengthening. All sessions were individually completed. Physical therapy sessions were 3 visits/week for the first 1-2 weeks, then 2 visits/week for 2 weeks, then 1 visit/week for 2 weeks, then 1-3 visits/month thereafter until 6 months. The HEP was 1 session/day for 6 months. Occupational therapy/rehabilitation nursing session were for 6 months. | **Low intensity multimodal exercise:**  Participants had returned home within 100 days of surgery. Physical therapy was supervised and comprised of mobility and transfer practice, and unspecified strengthening and flexibility exercise. Some participants received occupational therapy but no details about this are provided. The duration of treatment was determined by therapists providing usual care. | Subjective  Assessment of gait using 5 items from the gait component of the Performance Oriented Mobility Assessment  Objective  Time to walk 10 feet turn and walk back, timed ascent of a flight of stairs | 6 & 12 months post fracture |
| Tsauo et al. 2005  (Taiwan) | N = 54 (Int: 28; Con: 26);  Age = Int: 74.1 (12), Con: 71.9 (12.5);  Sex = Int: 3: 10, Con: 2: 10  *There were 54 participants enrolled in the study however age and sex were only reported for participants who completed follow-up. This data is present above. | Community | **Higher dose physiotherapy:**  Commenced the week of discharge from acute care, length of stay was 11.1 (4.2) days. Participants performed an unsupervised HEP of strengthening of the hip muscles and knee extensors, hip range of movement, balance and functional exercises. This was taught by a physical therapist during 8 home visits. The HEP was progressed by increasing the repetitions or by using resistance. Participants also received written material explaining their injury and exercise programme, and received weekly phone calls to improve exercise adherence. Participants’ mobility aids and home environment were also adapted. The HEP was individually completed, 1 session/day. There were 8 home visits over 12 weeks. | **Lower dose physiotherapy:**  Participants were advised to continue the unsupervised HEP provided during their acute care. No details on the content of this HEP are provided. This was individually completed. | Subjective:  Harris Hip Score^1^  Objective:  Gait speed | 1, 3, & 6 months post discharge |
| van Ooijen et al. 2016 (Netherlands) | N = 70 (Adaptability treadmill training: 24, Conventional treadmill training: 23, Con: 23);  Age = Adaptability treadmill training: 82.9 (6.5), Conventional treadmill training: 83.9 (5.5), Con: 83.3 (8);  Sex = Adaptability treadmill training: 8: 16, Conventional treadmill training: 9: 14, Con: 2: 21 | Inpatients | **Adaptability treadmill training and multimodal exercise:**  Baseline was median (range) 13 (7-65) days after fracture.  Adaptability treadmill training component:  Supervised treadmill walking that required participants to alter their step length and speed, and avoid obstacles, in response to visual projections on a treadmill.  Multimodal exercise component:  See adjacent column.  Typically 2 participants/session, sessions were 40 minutes but participants alternated exercise and rest resulting in 20 minutes of actual exercise. Participants completed 15 sessions of adaptability treadmill training and 15 sessions of multimodal exercise. | **Conventional treadmill training and multimodal exercise:**  Baseline was median (range) 13 (6-63) days after fracture.  Conventional treadmill training component:  Supervised treadmill walking that focussed on improving mobility quality, safety, endurance, and speed.  Multimodal exercise component:  See below.  Typically 2 participants/session, sessions were 40 minutes but participants alternated exercise and rest resulting in 20 minutes of actual exercise. Participants completed 15 sessions of conventional treadmill training and 15 sessions of multimodal exercise.  **Multimodal exercise:**  Baseline was median (range) 14 (7-79) days after fracture. Supervised lower limb strengthening, balance, functional movement and mobility exercise, and activities of daily living practice.  Typically 2 participants/session, sessions were 40 minutes but participants alternated exercise and rest resulting in 20 minutes of actual exercise, 5 sessions/week for 6 weeks, 30 sessions in total. | Subjective:  Performance Oriented Mobility Assessment, Functional Ambulation Category  Objective:  Timed Up and Go Test,  10m walk test, 10m walk test with obstacles, 10m walk test with cognitive task (the objective outcomes were not completed at final follow-up)  Mixed:  Elderly Mobility Scale | 6 weeks, 10 weeks, & 13.5 months |
| Williams et al. 2016  (UK) | N = 61 (Int: 29, Con: 32);  Age = Int: 80.9 (6.6), Con: 78.0 (8.3);  Sex = Int: 6: 23, Con: 9: 23 | Inpatients, community, and outpatients | **Behaviour change strategies, functional movement exercise, and usual care:**  Behaviour change strategies and functional movement exercise component:  Commenced soon after or before discharge from acute care, length of stay was 15.2 days. Participants were provided with a workbook explaining their injury and a goal setting diary to encourage unsupervised physical activity and activities of daily living practice. 6 sessions were provided after discharge. The content of these sessions was informed by the participants’ goals and therapists’ clinical judgement. Individually completed. The frequency was determined by therapists’ clinical judgement.  Usual care component:  See adjacent column. | **Usual care:**  Usual multi-disciplinary care provided by local health care services. This did not included the provision of written information. | Objective:  Timed Up and Go Test,  Fifty foot walk test | 3 months |
| Zidén et al. 2008  (Sweden) | N = 102 (Int: 48, Con: 54);  Age = Int: 81.2 (5.9), Con: 82.5 (7.6);  Sex = Int: 19: 29, Con: 12: 42 | Inpatient and community | **Behaviour change strategies, higher dose functional movement exercise, and functional training:**  Behaviour change strategies and functional exercise component:  During acute care participants’ self-efficacy was developed and goals were established. Participants also received an exercise programme. At home participants received physiotherapy visits that focussed on restoring mobility and improving self-efficacy, and occupational therapy that focussed on restoring independence with activities of daily living. Individually completed, frequency varied (mean of 5 visits (0.4), range 0-13) for a maximum of 3 weeks.  Lower dose functional movement exercise and functional training component:  Participants also received this intervention. See adjacent column. | **Lower dose functional movement exercise and functional training:**  Participants received supervised physiotherapy and occupational therapy to improve mobility, transfers, and activities of daily living performance. Mobilisation aimed to start within 48 hours post-operatively. A home visit was also performed if deemed necessary. Participants also received written information and advice about their condition and the importance of physical activity. Group-based and individually completed, every day until discharge. | Objective:  Timed Up and Go Test | 1 month  post discharge |
| Ankle fracture | | | | | | |
| Moseley et al. 2015  (Australia)  *Only females ≥ 50 years of age considered for this review* | N = 47 (Int: 23, Con: 24);  Age = NR for women over 50 sub-group  Sex = All females | Outpatients | **Resistance, balance, and flexibility exercise, and advice:**  Commenced immediately after removal of immobilisation, length of immobilisation was 45.3 (11.2) days.  Resistance, balance, and flexibility exercise component:  Supervised physiotherapy sessions and unsupervised HEP of progressively challenging high intensity lower limb strengthening, weight-bearing ankle range of movement, and single leg balancing exercises. Individually completed, suggested frequency of 2 sessions in week one, 1 session in weeks 2 – 4. The HEP was 1 session/day.  Non-weight bearing flexibility exercise and advice component:  See adjacent column. | **Non-weight bearing flexibility exercise and advice:**  Commenced immediately after removal of immobilisation, length of immobilisation was 47.9 (13.2) days. Participants received written information and advice on pain and swelling management, and returning to normal activities. Participants also received a HEP of non-weight bearing ankle range of movement exercises to complete unsupervised. Individually completed, session duration NR, 1 session only. The HEP was 2 sessions/day. | Subjective:  Lower Extremity Functional Scale, International Physical Activity Questionnaire (shot form)  Objective:  Gait speed | 1, 3, & 6 months |

Con = Control, HEP = Home Exercise Programme, Int = Intervention, N/A = Not applicable, NR = Not reported, UC = Unclear

Numbers are mean (SD) unless otherwise stated

^1^The Harris Hip score is not categorised as a Mixed Mobility Outcome as the objective component of this outcome does not assess mobility

^2^The location of the intervention is not clearly stated in the article however, as the intervention was administered in a group format by healthcare professionals, we have inferred it occurred in an outpatient setting.

^3^The modified Grimby Scale and 24 month follow-up time point were obtained from an additional report of this study: Turunen et al. 2017. Physical activity after a hip fracture: effect of a multicomponent home-based rehabilitation program—a secondary analysis of a randomized controlled trial. *Archives of physical medicine and rehabilitation*, *98*(5), pp.981-988.

^4^Information about the control group was obtained from Handoll et al. 2011. Interventions for improving mobility after hip fracture surgery in adults. *Cochrane Database of Systematic Reviews*, Issue 3. Art. No.: CD001704. DOI: 10.1002/14651858.CD001704.pub4.

**Definitions:**

- Setting of intervention: where supervision/advice was received (community, inpatient, outpatient)
- Follow-up is from randomisation/baseline unless stated otherwise
- Inactive control: if usual care for both groups has ceased at time experimental intervention was initiated, if control group received a HEP or referral to physio this is considered an intervention
